# White Matter Damage in Alzheimer’s Disease: Contribution of Oligodendrocytes

**DOI:** 10.2174/1567205020666221021115321

**Published:** 2022

**Authors:** Jinyu Zhou, Peng Zhang, Bo Zhang, Yuhan Kong

**Affiliations:** 1 Department of Rehabilitation Medicine, The First Affiliated Hospital of Chongqing Medical University, Chongqing-400042, China;; 2 Department of Neurology, The Second Affiliated Hospital of Chongqing Medical University, Chongqing-400010, China;; 3 Department of Basic Medicine, Chongqing Medical and Pharmaceutical College, Chongqing-401331, China

**Keywords:** Alzheimer’s disease, signaling pathways, oligodendrocytes, myelin sheath, central nervous system, white matter

## Abstract

Alzheimer’s disease (AD) is an age-related neurodegenerative disease seriously influencing the quality of life and is a global health problem. Many factors affect the onset and development of AD, but specific mechanisms underlying the disease are unclear. Most studies investigating AD have focused on neurons and the gray matter in the central nervous system (CNS) but have not led to effective treatments. Recently, an increasing number of studies have focused on white matter (WM). Magnetic resonance imaging and pathology studies have shown different degrees of WM abnormality during the progression of AD. Myelin sheaths, the main component of WM in the CNS, wrap and insulate axons to ensure conduction of the rapid action potential and axonal integrity. WM damage is characterized by progressive degeneration of axons, oligodendrocytes (OLs), and myelin in one or more areas of the CNS. The contributions of OLs to AD progression have, until recently, been largely overlooked. OLs are integral to myelin production, and the proliferation and differentiation of OLs, an early characteristic of AD, provide a promising target for preclinical diagnosis and treatment. However, despite some progress, the key mechanisms underlying the contributions of OLs to AD remain unclear. Given the heavy burden of medical treatment, a better understanding of the pathophysiological mechanisms underlying AD is vital. This review comprehensively summarizes the results on WM abnormalities in AD and explores the relationship between OL progenitor cells and the pathogenesis of AD. Finally, the underlying molecular mechanisms and potential future research directions are discussed.

## 1 INTRODUCTION

Alzheimer’s disease (AD) is an aging-related neurodegenerative disease clinically characterized by cognitive impairment and memory loss, which seriously affects the quality of life of patients [1]. Epidemiological surveys have shown that 44 million people worldwide suffer from AD. With the increase in the global population and aging, the prevalence of AD is expected to triple by the year 2050 [2-4]. The pathological mechanisms underlying AD are complex. Previous studies have shown that the main pathological features of AD are extracellular senile plaques composed of aggregated β-amyloid (Aβ) peptides, intracellular neurofibrillary tangles (NFT) containing hyperphosphorylated tau protein, Aβ cerebral amyloid angiopathy A, and neuronal loss caused by Aβ deposition on the vascular wall [5, 6]. In recent years, many studies have focused on amyloid levels in cortical and subcortical gray matter, based on the amyloid cascade hypothesis [7]. However, the neuropathological effects on white matter (WM), which are mainly caused by small vessel cerebral degeneration and inflammatory events, have not been fully understood and little attention has been paid to abnormalities of WM, such as demyelination and apoptosis of oligodendrocytes (OLs) [8-10].

Magnetic resonance imaging (MRI) findings have shown that patients with neurodegenerative diseases, including AD, have variably high WM signals, suggesting that WM changes may play an important role in altered cognitive function [11]. Of 24 patients with subcortical vascular cognitive impairment (SVCI) and 20 healthy subjects who underwent structural MRI, the results demonstrated that there were significant differences between the two groups in terms of WM volume and WM hyperintensities [12]. The cingulum bundle is widely recognized as one of the most significantly affected WM tracts in AD and mild cognitive impairment (MCI) [13, 14]. In a study that used a 3.0 Tesla MRI, the fractional anisotropy values obtained were significantly reduced in the WM (including the posterior part of the corpus callosum) and the anterior and posterior cingulate bundles in patients with AD compared to healthy participants [15]. Similarly, another study established that fatty acid (FA) levels in the anterior cingulate and splenium were lower in the AD group compared to the control group [16, 17]. These findings suggest that changes in WM may be useful in evaluating the progression of AD.

In the central nervous system (CNS), WM accounts for approximately 50% of the brain by volume [18]. The axons in the WM ensure the transmission of information between neurons to maintain normal neuronal functioning. OLs synthesize a special membrane, the myelin sheath, which wraps around axons in multiple layers to ensure rapid conduction of the action potential and ensure axonal integrity [19]. WM injury is characterized by progressive degeneration of the axons, OLs, and myelin sheaths in one or more regions of the CNS [20]. The main function of OLs in the CNS is to myelinate axons. Without myelin, the amplitude of action potentials decreases, their latency increases, and their conduction velocity decreases, which comprehensively demonstrates that myelination plays an important role in the process of electrical signal conduction [21, 22]. OLs are vulnerable to ischemic and pro-inflammatory changes in the environment, and their death triggers further demyelination far beyond the initial injury site, resulting in impairment of sensory, motor, cognitive, and autonomic nerve functions [23]. A clinical trial showed that the pathogenesis of AD is closely related to changes in the amount of myelin, while changes in the volume and microstructure of WM are also associated with brain degeneration, suggesting changes in WM and myelin formation are involved in the pathogenesis of AD [24]. However, despite the progress being made, the key mechanisms underlying the roles of OL progenitor cells and OLs in AD are still unclear.

Given the heavy burden of medical treatment for AD, a better understanding of the pathophysiological mechanisms underlying the disease is needed. Therefore, in this review, we comprehensively summarize the research findings on WM abnormalities in AD and discuss the relationship between OL and the pathogenesis of AD. In addition, we also discuss research progress toward elucidating the molecular mechanisms underlying AD and potential future research directions.

## 2 PATHOGENESIS AND RISK FACTORS FOR ALZHEIMER’S DISEASE

AD is an age-related neurodegenerative disorder characterized by chronic inflammation, synaptic loss, neuronal death, accumulation of insoluble protein aggregates and intracellular NFTs, and the deposition of extracellular amyloid plaques [25-27]. The accumulation of Aβ-amyloid and tau proteins in plaques and tangles is a central feature of AD [28]. In the pathogenesis of AD, Aβ aggregates are assembled from Aβ monomers into various unstable oligomers. Aβ aggregates are the main components of senile plaques and can cause neuronal and synaptic dysfunction. Although Aβ monomers in the body do not usually cause toxic reactions, research evidence shows that Aβ oligomers are neurotoxic [29, 30]. However, evidence shows that the Aβ amyloid aggregation of A amyloids is not sufficient alone to cause AD [31]. In recent years, clinical studies of drugs targeting amyloid beta protein (Aβ) in symptomatic patients with conventional (late-onset) AD have not been as effective as expected, making the validity of the amyloid hypothesis questionable [32, 33].

Tau is an important microtubule-binding protein (MAP), which is highly expressed in the CNS and is found in the axonal and synaptic regions of neurons [34, 35]. In the brain of a healthy person, tau protein binds to nerve microtubules to provide stability and allow axons to transport nutrients and transmit information [36]. However, in AD, tau protein becomes hyperphosphorylated, leading to a decrease in its ability to bind to nerve microtubules and the resulting synaptic and axonal damage and neuronal degeneration [37]. Postmortem studies in patients with AD have indicated that cortical tau pathology is associated with WM microstructural integrity alterations detected by diffusion MR (dMRI) [34]. Furthermore, a recent study showed that high tau deposition was associated with a lower axonal packing density and a high degree of radial diffusion freedom [35]. Currently, a popular view is that Aβ and tau have a synergistic effect in AD [28, 38]. *In vitro* experiments have shown that tau deletion prevents Aβ-induced neuronal cell death, while re-expression of the knocked-out tau gene in neuronal cells restores Aβ-induced neurotoxicity [39]. Furthermore, reduction of tau expression was found to protect neurons against Aβ-induced axonal transport defects, consistent with the results of previous studies [39, 40].

Many factors are known to affect the appearance and development of AD, such as aging, genetic factors, head injuries, vascular diseases, infection, and environmental factors [25]. Among them, aging is the most important risk factor, and most patients with AD start to show signs of the disease after the age of 65 years [41]. Research indicates that loss of function of the Δ113p53 isoform of the aging-related gene p53 may cause long-term oxidative stress and aggravate the development of AD [42]. Because aging is a complex and irreversible process, it is accompanied by loss of brain size and weight and of neuronal synapses. Neurons in the brain of patients with AD exhibit many markers of aging-related processes, including genomic instability, shortening of the telomere, epigenetic changes, and mitochondrial dysfunction [43]. Furthermore, the levels of DNA methylation and protein acetylation of some proteins change during the aging process, accelerating pathological processes in AD [44, 45]. Moreover, the aging of glial cells such as astrocytes and OL also contributes to the pathogenesis of AD. Factors such as viral and bacterial infections, metal ion levels, lifestyle habits, intestinal flora, eating habits, and environmental factors also affect the incidence of AD.

### 2.1 Changes in White Matter in Alzheimer’s Disease

In recent years, the massive research demonstrated that WM in the brain had become an important focus of study to understand the mechanisms underlying AD (Table **1**). WM is composed of concentrated myelinated axon fibers that extend from neuronal and glial cell bodies. The WM includes myelinated axons and different types of glial cells, including myelinated OLs, OL progenitor cells, astrocytes, and microglia [46, 47]. MRI results have shown that changes in WM are closely related to abnormalities in lifestyle and in the sensorimotor and cognitive domains [46]. WM tracts in the brain serve primarily as functional connections and process signals transmitted between neurons. Therefore, impairment of WM integrity during aging can significantly affect cognitive function [48, 49]. However, WM degeneration in AD changes with the progression of the disease [50]. Furthermore, a study that specifically investigated brain aging in female subjects interpreted ketones production by myelin lipid catabolism during the aging process as a system-level adaptive response to meet the energy demands of the brain, further elucidating the underlying causes of aging. Therefore, identifying the initiating factors and mechanistic pathways of WM catabolism in female subjects provides potential therapeutic targets for preventing and treating demyelinating brain diseases, such as AD [51].

The question of whether the mechanisms underlying WM aging are related to sex has been directly addressed in a previous clinical study [52], which investigated the relationship of WM integrity in the brain with age and sex based on a large sample (857 healthy subjects) (Table **1**) [53-63]. The results suggested that in certain areas of the brain, the number of remyelinated axons increases with age; however, no sex-based difference was found with respect to WM aging. In AD, it is not clear whether the degree of damage from WM is related to sex [52]. Lauren *et al.* [51] showed that in the AD model of natural aging, the accumulation of lipid droplets and the production of ketones decrease concomitantly. Furthermore, electron microscopy and lipid analysis confirmed myelin degeneration, suggesting that ketone bodies produced by myelin lipid catabolism could provide a potential therapeutic target for the prevention and treatment of demyelinating diseases, such as AD and multiple sclerosis [51]. Furthermore, Owen *et al.* [53] examined the superficial WM of the whole brain in patients with AD and in healthy control subjects. Their results showed that, compared to the control group, the diffusion rate increased in the superficial WM of the whole brain of patients with AD. These important findings suggest that superficial WM damage can cause axonal atrophy (increased axial diffusion) and myelin damage and that superficial WM throughout the brain is vulnerable to AD [53].

WM inflammation, as an important contributor to inflammation, has attracted increasing attention in the literature. Neuroinflammation in WM may be used as an early marker to predict cognitive decline during aging. The results of a recent study suggested that inflammation of the WM is an early pathological change during the progression of AD [8]. Furthermore, brain tissue analysis has shown that broad areas of WM, including the corpus callosum, pili, internal capsule, cingulate, anterior commissure, and optical tract, are inflamed during the early stages of AD [8]. Similarly, another study found that the level of neuroinflammation in microglial-induced WM was increased in patients with AD compared to younger control subjects [9]. An increasing number of approaches have been applied to the study of neurodegenerative diseases to provide valuable information for the diagnosis of these diseases. For example, a recent study used a superresolution imaging technique known as stochastic optical reconstruction microscopy (STORM) to analyze human brain slices, helping to reveal the potential mechanisms underlying common nervous system diseases [54]. Therefore, the use and development of novel neuroimaging technologies play a key role in the diagnosis and treatment of neurodegenerative diseases.

### 2.2 Myelin Damage in Alzheimer’s Disease

Increasing evidence has shown that abnormalities of the WM are closely related to many neurological diseases and that myelination is one of the main processes that regulate WM function [47]. The brain undergoes a long-lasting myelination process, although the speed at which neuronal processes grow varies (peaking in middle age) to maintain the transmission speed of the action potential. The myelin sheath provides discontinuous insulation along the axon, contributing to the conduction of saltatory impulses and the regulation of physiological functions in the brain. When the rate of myelin formation slows or the fragility of the myelin sheath increases, AD-related degenerative phenotypes begin to appear [64]. Remyelination is a natural physiological process that prevents nerve degeneration and restores nerve function [64]. Myelin injury refers to the reduction of processes of myelin loss and generation around brain axons, with the most common form being demyelination. In demyelinating diseases, insufficient myelin regeneration is closely related to the progression of the disease [65-67]. Myelin injury and loss have an important impact on the progress of AD; therefore, improving and protecting normal synaptic and myelin structure are key therapeutic goals in the treatment of AD [68, 69].

Leucine-rich repeat protein 1 and immunoglobulin domain containing protein 1 (LINGO-1) is an attractive target for myelin regeneration in the CNS [70]. Wu *et al.* [71] found that abnormal increases in the number of myelinated axons and the thickness of the myelin sheath, and the decrease in myelin basic protein (MBP) expression in 1-month-old 5XFAD mice (co-expressing human APP and presenilin 1 with five familial AD mutations, including APP K670N/M671L + I716V + V717I and PS1 M146L + L286V) was related to spatial memory impairment. Furthermore, *in vivo* injection of the LINGO-1 antibody reversed myelin injury in some of the brain regions related to memory impairment. These results indicated that myelin injury is related to memory impairment in patients with AD, suggesting that increasing myelin regeneration could be a potential therapeutic strategy in the early stage of AD [71]. In addition, another study has shown that neuritis has a particularly important impact on myelin regeneration [72]. Although an active inflammatory response is common in CNS diseases and can damage myelin sheaths, the inflammatory process also has a positive impact on the progression of the OL lineage, the clearance of OL myelin fragments, the metabolism of OL, and myelin repair [72]. Several autopsy and imaging tests have found that patients with AD have varying degrees of myelin loss [73-76]. At the same time, single-cell and spatial transcriptome analyses have revealed that expression of genes involved in myelin regeneration is up-regulated in the brains of patients with AD [77-79]. The above studies indicate that there is sustained and extensive myelin damage during the progression of AD.

#### 2.2.1 Myelin Damage in Alzheimer’s Disease: Abnormalities of Oligodendrocytes

##### 2.2.1.1 Oligodendrocytes and Neurons and ApoE

In the brain, cholesterol plays a key role in synaptic function. It helps maintain a sufficient curvature of the membrane to promote soluble NSF attachment protein receptor (SNARE)-mediated membrane fusion, of which Apolipoprotein E (ApoE) is one of the main components [47]. Cholesterol depletion significantly reduces synaptic transmission, which can be reversed by reloading cholesterol [80]. Cholesterol has been shown to play a key role in myelination. A marked reduction in OL myelination is caused by inhibiting cholesterol synthesis capacity in OLs. Furthermore, it was also observed in the OL lineage during the remyelination phase after axonal injury to elevate the expression of genes associated with cholesterol biosynthesis (*i.e.,* Hmgcs1, Fdps, and Fdft1) [81, 82]. OLs are highly specialized cells of the CNS, whose main function is to form myelin [83]. In recent years, the impact of the loss of OL and myelin dysfunction on various neurodegenerative diseases, including AD, has become increasingly evident [84]. *In vitro* findings showed that ApoE expression in OLs increases when neurons are mechanically damaged, and OLs can secrete ApoE into the surrounding environment [85]. Compared to wild type mice, the density of OPCs in the molecular layer DG of p301 mice with silenced ApoE gene expression (ApoE KO) increased, suggesting the potential role of proliferating OPCs in the repair of neurodegenerative damaged myelin and indicating the internal regulation of ApoE/LDLR on myelin formation, which may be partly due to regulation of OPCs [86].

Wu *et al.* [61] reported changes in myelin morphology and OL development in the hippocampus of 2-month-old APP/PS1 mice and found that neurons are essential for OL development and myelination. The authors also found that type III regulatory protein 1 was up-regulated, further indicating that hippocampal changes in myelin morphology and OL development occurred before behavioral deficits and most of the other pathological features observed in mice [80]. Furthermore, Dong *et al.* [73, 81] evaluated myelin injury in the corpus callosum and temporal lobe of 6-month-old APP/PS1 mice. Their results showed that myelin levels and expression of OL monocarboxylate transporter 1 (MCT1) decreased in APP/PS1 mice at 6 months, indicating that OL maturation was impaired and that OL dysfunction and early demyelination may lead to the progression of AD.

##### 2.2.1.2 Tau Protein in OLs

In OL, tau protein plays a key role in myelination [87]. Animal studies have shown that OL tau protein can promote myelination by inducing the basic protein myelin and promoting the growth of OL processes that wrap around axons [88, 89]. In transgenic mice expressing Δtau, OLs showed reduced myelination [90]. Furthermore, mature s of MBP / Olig2 OLs increased twofold in 2-month-old P301S-htau mice compared to WT mice [91]. Given that loss of oligodendroglial tau function could weaken myelination, there may be a crucial link between pathological tau aggregates in OLs and AD degeneration [82].

##### 2.2.1.3 Oxidative Damage and Oligodendrocytes

Oxidative damage has also been shown to contribute to the toxicity of Aβ in OLs. Studies by Yasuyuki *et al.* [83] found that the expression of growth arrest and DNA damage-inducible protein 34 (GADD34) in neurons and in OLs was significantly increased in the brains of AD-model mice. Compared to control mice, the level of GADD34 expression in APP transgenic mice increased in the early stage of the disease, suggesting that oxidative stress exerted by Aβ oligomers is more correlated with GADD34 expression in OLs than that in neurons [83]. Mature OLs seem to rely on the glycolysis process to produce energy, but even with sufficient oxygen, OLs consume a large amount of lactic acid to provide the energy required for myelination and to further maintain the long-term integrity of axons [84]. A recent basic and clinical experimental study found nucleotide-binding oligomerization domain-like receptor protein 3 (NLRP3)-dependent inflammatory damage in mature OLs in both patients with AD and in AD-model mice, accompanied by demyelination and axon degeneration [85]. A further study of the underlying mechanism indicated that excessive activation of dynamin-related protein 1 (Drp1, a mitochondrial fission guanosine triphosphatase) in mature OLs inhibited glycolysis and activated NLRP3 inflammasomes by inhibiting hexokinase 1 (HK1), proving that the Drp1/HK1/NLRP3 signaling axis plays a key role in maintaining the metabolic homeostasis of mature OLs [85, 86]. These results provide novel insights into the mechanisms underlying WM degeneration in AD, thus identifying potential new approaches for therapeutic interventions [85, 92, 93].

**Fig. (1):**
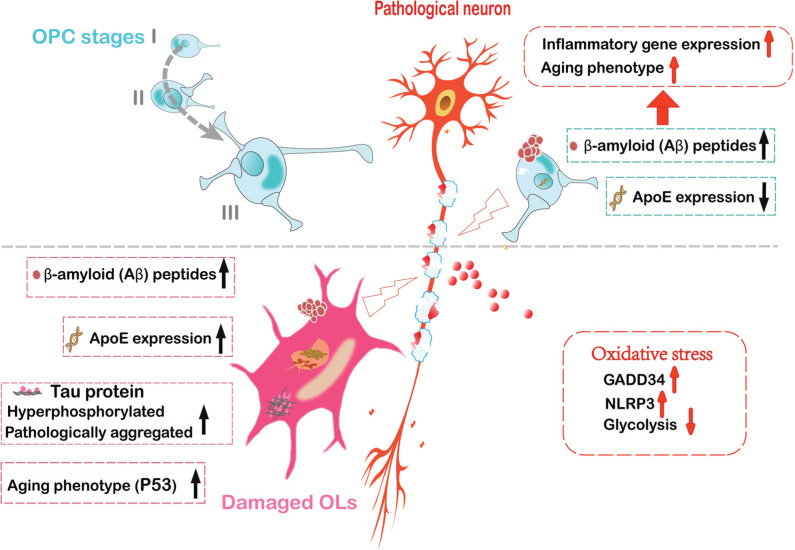
Pathological changes of myelin sheath play an important role in the pathogenesis of AD. The morphological changes of OPC were characterized by cell contraction and small nucleus in the early stage, and cell hypertrophy and increased branching in the late stage. In OPC, AD risk gene ApoE is specifically inhibited. In addition, Aβ plaques accumulate outside OPC cells, which directly acts on OPC to induce aging phenotypes and increase expression levels of inflammatory genes, leading to neuronal damage. The morphologic changes of OLs showed smaller nuclear than the normal. In OLs, the expression of ApoE and p53 increased. Pathological Tau aggregation and Tau hyperphosphorylation occurred in OLs, leading to OLs senescence and apoptosis. Furthermore, the oxidative stress results in OLs DNA damage, increased NLRP3 inflammasomes and GADD34, as well as decreased glycolysis.

The size of the nucleus in OLs has been reported to be smaller in AD mice than in controls. Furthermore, some OL nuclei show oxidative damage to DNA, while other OLs have senescent phenotypes and increased expression of the senescence stress marker p53, suggesting that OL apoptosis and aging play an important role in the progression of AD [87, 88, 93]. In addition, regulators of CNS development play a significant role in myelin formation and cell processes. Studies have shown that fibroblast growth factor (FGF) receptor signaling in OL inhibits myelin degeneration and myelin decomposition in AD mice and in patients with AD, as well as improving or restoring the pathological and clinical symptoms of cognitive decline. Therefore, FGF could be used as an intervention and potential therapy for myelin decomposition and cognitive decline in AD [89]. The above studies suggest that preventing OL loss and promoting myelin regeneration in AD may contribute to promising therapeutic approaches in the future (Fig. **1**).

#### 2.2.2 Myelin Damage in Alzheimer’s Disease: Abnormalities in Oligodendrocyte Progenitor Cells

OL progenitor cells (OPCs) are distributed throughout the CNS and represent a group of adult migratory and proliferative progenitor cells that can differentiate into OLs; only 5% to 8% of glial cells are OPCs, most of which are distributed in the WM [90, 91]. OPCs, which can be recognized by the expression of neuron glial antigen 2 (NG2) proteoglycans, are sometimes called NG2 cells or NG2 glia, and are the main type of proliferating cells in the brain [94, 95]. Up-regulation of NG2 cells in patients with AD can represent an increase in OL production to compensate for the loss of OL in the disease [96]. During the development of the CNS, OPCs proliferate and migrate to their destinations before finally myelinating axons after differentiation into mature OLs, a process that is strictly regulated [65]. OPCs are mobilized in response to neuronal damage and demyelination, proliferate, and move to the damaged site [97]. In particular, OPCs exhibit increased signs of the senescence marker Cdkn2a in the aging brain [98, 99]. Age-related changes in OPC are related to the reduction in myelination in AD, but the specific molecular mechanisms that affect OPC in AD are rarely studied [100]. Zhang *et al.* [100] found that Aβ in the brains of patients with AD and in AD model mice act directly on OPC to induce aging phenotypes and increase inflammatory gene expression, leading to neuronal damage. Long-term intermittent aging treatment was found to reduce Aβ expression in plaque-associated pro-inflammatory cytokines [100]. During this specific time window (6–8 months), OPC proliferation and differentiation in APP/PS1 mice increased, suggesting that improvement in myelin aberrations is caused by the OPC-mediated repair mechanisms [101].

**Fig. (2):**
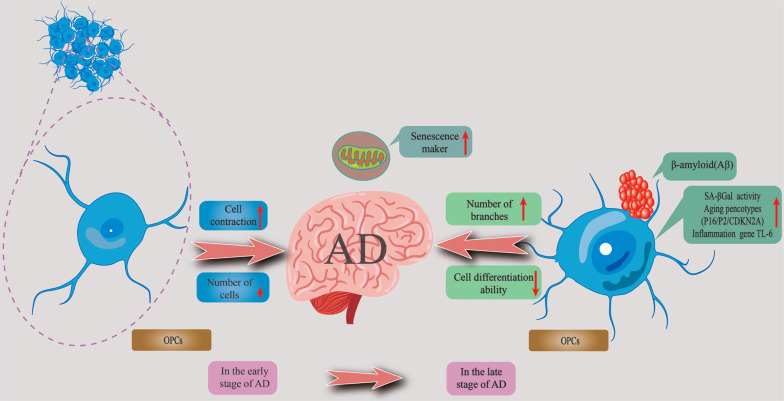
The change to OPCs is a potential factor in the pathological progression of AD. OL progenitor cells (OPCs) undergo complex morphological changes in AD, characterized by obvious cell contraction, declined number of cells. In the late stage, OPCs showed obvious hypertrophy and increased branching.

To study cellular diversity and disease-related cellular changes in the structure of the AD network, Grubman *et al.* [77] used a single cell RNA sequencing map (DroNc-Seq) of the entorhinal cortex of patients with AD to reveal transcriptional changes of specific cell subsets. Their results showed that the AD risk gene apolipoprotein E (APOE) was specifically inhibited in OPCs and astrocyte subsets, while also being up-regulated in specific microglial subsets [77]. A recent study provided strong evidence on the effects of AD on PCB by studying the CA1 region of the hippocampus. The results of the study showed that the number of OPCs decreased prematurely at 9 months in APP/PS1 mice, while OPCs showed cell contraction and increased branching at 14 months. These findings are consistent with evidence of disruption of myelin formation in AD-model mice reported in previous studies [80, 102]. Furthermore, the authors found that the number of OPCs in APP/PS1 mice decreased significantly at 9 months, with OPCs showing contraction and altered fiber morphology at 14 months. These were characterized by an overall contraction of OPC processes and an increase in the number of branching processes, indicating morphological malnutrition. These findings suggest that changes to OPCs are a potential factor in the pathological progression of AD [66]. OPCs undergo complex morphological changes in AD, characterized by obvious cell contraction in the early stage of AD and obvious hypertrophy in the late stage. These results show that complex changes in OPCs are important markers of early and late AD-like pathology and maybe the main factor contributing to myelin loss. Therefore, this study emphasizes the importance of OPCs in the pathogenesis of AD [94]. These findings suggest that changes to OPCs are a potential factor in the pathological progression of AD (Fig. **2**).

#### 2.2.3 Signaling Pathways Involved in Myelin Damage Injury in AD

A wide array of signaling mediators and their interactions play vital roles in developmental disorders and age-related neurodegenerative diseases [103]. In the pathogenesis of AD, a variety of signaling pathways regulate myelin formation. Autophagy is often studied in CNS neurons, but less frequently in glial cells. Autophagy plays a key role in the maturation and structural plasticity of OLs [104, 105]. In one study, mice with conditional OPC/OL-specific deletion of autophagy-related gene 5 (ATG5) died 12 days after birth [104]. Further analysis showed that OPCs increased apoptosis, decreased differentiation, and decreased myelination. In contrast, increased autophagic activity led to increased myelination, suggesting that autophagy plays a key role in regulating myelination [104]. Current evidence indicates that the nuclear factor erythroid 2-related factor 2 (Nrf2) pathway is highly important in the regulation of neurodegenerative diseases [106-108]. Studies have found that the sequestosome 1 (p62)–Kelch-like ECH-associated protein 1 (Keap1)/Nrf2 positive feedback loop is the bridge between the Nrf2 pathway and autophagy, and plays an important role in the occurrence and development of AD [109, 110].

Changes in a variety of signaling pathways, such as the Wnt signaling pathway [110, 111], the PI3K/Akt signaling pathway [112], and the AMPK/SIRT1 signaling pathway [113, 114] have previously been reported in studies of aging and neurodegenerative diseases, including AD. Wnt signaling is involved in the formation, plasticity, and maintenance of synapses [115]. Studies have shown that the typical Wnt antagonist Dickkopf-1 (DKK1) is upregulated in the brains of patients with AD and in AD mouse models and that restoration or enhancement of Wnt signaling can protect cells and synapses from Aβ toxicity and improve AD pathology [116]. Furthermore, the expression of PI3K, Akt, mTOR, p-PI3K, p-Akt and p-mTOR in 6-month-old APP/PS1 transgenic mice were found to decrease over time. When the mTOR signaling pathway was blocked, myelin-related protein expression levels were reduced, resulting in a reduction in- sheath formation [117]. The regulatory roles of many signaling molecules in AD have yet to be identified; therefore, more studies are needed to clarify the relationships of these signaling proteins with the development and regeneration of myelin in patients with AD.

## 3 DISCUSSION

The pathological mechanisms underlying AD are complex, and our limited understanding of the molecular and cellular changes in AD has hindered the identification of therapeutic targets. Alteration in WM is a pivotal event in AD and contributes to its pathogenesis [118]. Substantial evidence indicates that myelin and OLs are obviously significantly associated with AD-related pathology [67].

A recent study analyzed cell nuclei from the brains of patients with AD and of healthy controls using mononuclear RNA sequencing and found more than 2000 differentially expressed genes [119]. Further subgroup analysis conducted on single cell types identified nine subpopulations of OL in the brains of healthy control and AD subjects. The results showed that in AD, the number of mature myelinated OLs decreased, while the number of remyelinated OLs increased. The loss of myelinated OL subsets indicates the disruption of OL maintenance in AD, but it is not yet clear why myelinated regenerated OLs cannot completely supplement and improve the demyelination observed in AD [119]. Taken together, these findings clarify the potential to improve myelination as a treatment strategy to prevent and improve AD and will be an important focus of future research.

Epigenetic regulation, including DNA methylation, chromatin remodeling, and histone post-translational modification are of great significance to human health. In particular, DNA methylation has been investigated regarding its association with AD [120]. DNA methylation and histone modification can directly or indirectly influence the activity of tau-related kinase genes, promoting the occurrence and development of AD [121]. Furthermore, a recent study showed that m6A modification is involved in OPC proliferation and myelination [122]. This suggests that future research focusing on epigenetic modification of OLs could provide a novel direction for the development of effective treatment strategies for myelination-related diseases, such as AD [122]. Therefore, it is necessary to understand epigenetic modification mechanisms underlying AD-related pathologies in association with changes to the WM to provide new insights for the development of drug targets. Myelin degeneration and loss of WM resulting from the death of OLs is usually one of the first changes in the brain of patients with AD, with many potential causes responsible for the loss of myelin [66]. Metabolic stress in OLs triggers inflammation and OL injury that culminates in demyelination, white matter degeneration, and cognitive impairment in models of AD [85]. Therefore, it is necessary to evaluate the proliferation, apoptosis, and metabolic changes of OLs to better understand the pathogenesis of AD. Furthermore, additional study is required to better identify targets promoting myelin regeneration and the signaling molecules acting on these processes to develop additional diagnostic markers and identify novel therapeutic targets.

## CONCLUSION

AD is one of the most common neurodegenerative diseases, characterized by neurological dysfunction and cognitive impairment. During the prevention, diagnosis, and treatment of AD, a growing number of studies have reported abnormalities in WM structure; however, research into the underlying mechanisms of such WM abnormalities is still relatively lacking. Additionally, there is still no effective treatment for AD available in the clinic. OL loss and myelin damage that characterize AD pathologically are not only secondary effects of neuronal degeneration but are also involved in the early pathological manifestation of AD. Previous studies have shown that age-related decrease in myelination accelerates the progression of AD, suggesting that myelination plays a key role in the occurrence and development of AD. Although we have an increasingly detailed understanding of OL maturation in the context of normal development, we still have little understanding of how processes, including apoptosis, proliferation, and differentiation in OLs contribute to the progression of AD. Therefore, it is important to better understand the relationship between myelin regeneration and AD, and to clarify the pathophysiological and molecular mechanisms underlying the association between OLs and AD. To address this, future research investigating the mechanisms of abnormal myelin formation in AD should pay closer attention to the molecular mechanisms of myelin formation, as well as the molecular processes and epigenetics in OLs. Such studies could lead to a breakthrough in the development of new therapies for AD in the clinic.

## Figures and Tables

**Table 1:** Changes in white matter in Alzheimer’s disease.

**Author**	**No. of Patients**	**Changes in White Matter**	**Refs.**
Phillips *et al.*	AD (N = 44), Healthy controls (N = 47)	Increase in the axial and radial diffusivity across the superficial white matter.	[53]
Mito *et al.*	AD (N = 49), MCI (N = 33), Healthy controls (N = 95)	White matter tracts, reductions in fiber density and cross-section.	[55]
Catheline *et al.*	Young controls (N = 15), Old controls (N = 15), AD (N = 15)	Mean FA values at cingulum bundle and mean number of fibers significantly declined in AD patients.	[56]
Choo *et al.*	AD (N = 19), MCI (N = 19), Healthy controls (N = 18)	AD and MCI patients showed a significant decrease of FA in the posterior cingulate cingulum.	[57]
Toniolo *et al.*	AD (N = 50), Healthy controls (N = 25)	AD patients showed a lower fractional anisotropy and a higher radial diffusivity in the middle cerebellar peduncle and the superior cerebellar peduncles.	[58]
Li *et al.*	AD (N = 21), MCI (N = 20), Healthy controls (N = 25)	AD patients showed a serious damage both white and gray matter of the limbic system networks.	[59]
Bozoki *et al.*	AD (N = 21), MCI (N = 23), Healthy controls (N = 16)	Fornix FA and volume were reduced in MCI and AD patients.	[60]
Huang *et al.*	AD (N = 26), MCI (N = 11), Healthy controls (N = 24)	The FA, mean diffusivity, axial diffusivity and radial diffusivity of 30 major cerebral white matter tracts are significantly correlated to cognitive functions in AD patients.	[61]
Lin *et al.*	AD (N = 9), MCI (N = 8), Healthy controls (N = 15)	Significantly smaller FA values were found in the posterior and inferior segments of left cingulum bundle and the anterior segment of right cingulum bundle of the AD patients.	[62]
Fieremans *et al.*	AD (N = 14), MCI (N = 12), Healthy controls (N = 15)	White matter tract integrity metrics in the body of the corpus callosum were strongly correlated with processing speed in amnestic mild cognitive impairment.	[63]

**Abbreviations:** AD: Alzheimer’s disease, MCI: Mild cognitive impairment, FA: Fractional anisotropy.

## LIST OF ABBREVIATIONS

AD: Alzheimer’s Disease
WM: White Matter
OLs: Oligodendrocytes
CNS: Central Nervous System
MRI: Magnetic Resonance Imaging
LINGO1: Leucine-Rich Repeat and Immunoglobulin Domain-Containing Protein 1
MBP: Myelin Basic Protein
MCT1: Monocarboxylate Transporter 1
GADD34: DNA Damage-Inducible Protein 34
Drp1: Dynamin-Related Protein 1
OPCs: Oligodendrocyte Progenitor Cells
NG2: Neuron Glial Antigen 2
Nrf2: Nuclear Factor Erythroid 2-Related Factor 2
ApoE: Apolipoprotein E
